# Using manual versus mechanized glide path instruments and ProTaper Gold versus ProTaper Next systems in curved canals: micro-CT study

**DOI:** 10.1590/1807-3107bor-2024.vol38.0006

**Published:** 2024-01-05

**Authors:** Breno Nappi VENTURA, Giulio GAVINI, Elaine Faga IGLECIAS, Laila Gonzales FREIRE, Celso Luiz CALDEIRA

**Affiliations:** (a) Universidade de São Paulo – USP, School of Dentistry, Department of Operative Dentistry, São Paulo, SP, Brazil.; (b) Universidade Paulista – UNIP, Department of Endodontics, São Paulo, SP, Brazil.

**Keywords:** Root Canal Preparation, Dental Instruments, Molar, X-Ray Microtomography

## Abstract

The aim of this study was to evaluate the root canal shaping effect of ProTaper Gold (PTG) versus ProTaper Next (PTN) instrumentation systems, and of a manual #15 K-type file (K15) versus the ProGlider (PG) mechanized instrument for glide path creation, in severely curved mesial canals. Twenty-four mandibular molars with two separate mesial canals were anatomically matched using computed tomographic scanning, and then divided into two groups (n=12) according to the glide path instrument used, either K15 or PG. In all teeth, the PTG system was used to prepare the mesiobuccal canal, and the PTN, the mesiolingual canal. The teeth were scanned by computed microtomography, before and after root canal preparation, and the values of the initial volume, final volume, volumetric variation, untouched walls, and canal transportation variables were determined. The data were analyzed using the two-way ANOVA test, and the Tukey test for multiple comparisons. There was no significant difference among the study groups regarding volumetric variation or root canal transportation, either in the cervical, middle or apical thirds, or in the entire root canal (p>0.05). In the apical third, the percentage of untouched walls was significantly higher in groups using K15 than in those using PG (p<0.05), namely 33.144% and 23.285%, respectively, irrespective of the instrumentation system. In the other regions, there was no difference between K15 and PG regarding this variable. It was concluded that PG was associated with a lower rate of untouched walls in the apical region than K15.

## Introduction

Root canal shaping is an essential step to ensure the effectiveness of subsequent endodontic treatment procedures, including chemical disinfection and root canal filling. The complex anatomy of the root canal system (RCS) and the inherent limitations of mechanized nickel-titanium (NiTi) systems pose challenges to accomplishing this step, especially in severely curved root canals.^1^ In recent years, mechanized instruments have undergone changes in their design, in the thermal and surface treatments used in their production, and in the kinematics used to drive them.^2,3^ Heat treatments increase instrument flexibility and reduce their cyclic fatigue.^4^

However, the fracture rate of instruments^5^ and the level of canal transportation they promote are greater in severely curved canals, owing to (a) the excessive force applied to the instrument in the apical direction,^6^ (b) the large area of contact between instrument and canal walls, and (c) the fact that the cross-section of teeth with severely curved canals may be smaller than the tip diameter of the instrument used.^7^

In this respect, prior creation of a glide path with fine-caliber instruments can be better recommended to reduce the torsional stress applied to the instruments used in the subsequent root canal shaping stage, thus increasing instrument performance and working lifespan.^8^ Another advantage to creating a glide path is the enhanced accuracy it provides in determining the working length.^9^ On the other hand, the type of instrument used to create a glide path can interfere with the resulting endodontic preparation; in this respect, some authors have observed comparatively higher levels of canal transportation when the glide path is created with stainless steel files.^10^

The ProGlider mechanized system consists of a single file to perform the glide path maneuver in continuous rotation. The instrument is made of M-Wire alloy, which gives it considerable flexibility, and it has a square cross-section with four cutting edges. Its tip diameter is 0.16 mm, and its taper is progressive (2%–8.5%), thus favoring preliminary enlargement of the root canal cervical and middle third regions, and the subsequent RCS shaping procedure.^11,12^

Other changes to endodontic instruments designed to improve preparation quality include the kinematics used to drive them. Standing out among these advances is the eccentric rotary movement produced by instruments whose centers of gravity and rotation are shifted. This configuration provides greater cutting efficiency, and enhanced displacement of dentin chips in the coronal direction, hence reducing the risks of both instrument fracture,^13^ and root canal transportation.^4^

Many 2D methodologies have been used to assess how well instruments produce well-centered preparations and low levels of canal transportation.^14-16^ In contrast, microcomputed tomography (micro-CT) is a non-destructive method that can be combined with software analyses to assess root canal transportation three-dimensionally in extracted teeth.^17^ Several authors have evaluated transportation at different root canal levels using micro-CT.^18-20^ Gagliardi et al.^4^ analyzed the behavior of this variable along the entire length of curved canals, but not in canals with severe curvatures (above 40°).

Severe curvatures and anatomical variations can pose a significant challenge to performing antisepsis of the endodontic space. In infected teeth, bacteria can persist not only in hard-to-reach areas, such as isthmuses, ramifications, and dentinal tubules, but also in oval/flat or C-shaped root canal extremities that instruments fail to touch, and where instruments tend to promote a rounded shape effect.^21^

Following the latest manufacturing trend, instruments of the Protaper Next system (a progression from the previous Protaper Universal system) are made of the M-wire NiTi alloy. Their off-center rectangular cross-section provides eccentric rotation, i.e. a rotation that takes place outside the center of mass of the instrument. This feature means that friction with the canal walls is created only by two cutting edges, whereas the other two edges work freely in the canal, thus reducing the risk of instrument torsion or fracture.^22,23^ Another recently introduced system was ProTaper Gold. Its instruments have exactly the same shapes, sizes, tapers, and cross-sections as those of the Protaper Universal system; however, according to the manufacturer, the metallurgical properties of the alloy used in their production increase their flexibility and resistance to cyclic fatigue.

Some studies have evaluated the shaping ability of mechanized rotary systems, and the levels of canal transportation they cause in severely curved molars;^13,24-26^ however, to date, no study evaluated the influence of glide path creation on the root canal preparation obtained with these systems. Thus, the aim of the present study was to conduct a three-dimensional assessment of the canal shaping effect and level of canal transportation promoted by the ProTaper Gold (PTG) versus ProTaper Next (PTN) instrumentation systems, and by a #15 K-type manual file (K15) versus the ProGlider mechanized instrument (PG) for glide path creation, in severely curved mesial canals of mandibular molars. The null hypothesis was that there would be no differences, either between the instrumentation systems or between the glide path creation instruments, with respect to canal shaping and canal transportation.

## Methodology

### Tooth selection and specimen preparation

This study was approved by the local research ethics committee (approval no. CAAE: 60535916.8.0000.0075). All of the tests related to specimen preparation and root canal shaping were performed on the same day, in the research laboratory of the Department of Restorative Dentistry, School of Dentistry, University of São Paulo, and were conducted under the same temperature condition, namely 22°C, which was controlled by means of an air-conditioning unit.

The sample size was calculated by selecting the ANOVA test from the F-test family, and setting an alpha error of 0.05, a beta power of 0.8, and an N_2_/N_1_ ratio of 1 (G* Power v 3.1; Heinrich Heine, Universität Düsseldorf). A total of 10 specimens per group was found to be the optimal size to detect significant differences. The sample size was increased by 20% considering the risk of instrument fracture.

Thus, twenty-four mandibular molars provided by the tooth bank of the university where the study was conducted were scanned with a cone-beam computed tomography scanner (I-Cat Imaging Sciences International, Hatfield, PA, USA) to ensure tooth selection according to the inclusion criteria: intact pulp chamber with no carious lesions, cracks, resorption, or previous endodontic treatment, complete root formation with type IV mesial root canals, according to Vertucci’s classification (independent canals and foramina), and mesial root canals with severe curvatures (between 40° and 68°^27^).

Then, the occlusal surfaces of the teeth were sectioned with a precision cutter (Isomet 1000; Buehler, Lake Bluff, USA) to standardize the specimens at 17 mm. Coronal access was performed with #1014 diamond burs (KG Sorensen, São Paulo, Brazil) and Endo Z burs (KG Sorensen). The mesial canals were passively explored with a #10 manual file (Dentsply Maillefer, Ballaigues, Switzerland) until the instrument tip was visualized through the apical foramen under an operating microscope (OPMI PROergo; Zeiss, Jena, Germany) at 8x magnification. The canals whose patency was not achieved with a #10 manual file were excluded from the experiment. The working length (WL) was established at 1 mm short of the apical foramen.

### Root canal preparation

The 24 specimens (48 root canals) were matched to create 4 groups of 12 roots, as described below, based on the three-dimensional morphologic aspects of the mesial canals, and divided according to the glide path creation instrument, either K15 or PG, and the instrumentation system used, either PTN or PTG. The PTG system was used to prepare the mesiobuccal canal, and the PTN, the mesiolingual canal, in all the teeth. The degree of homogeneity with respect to canal curvature was confirmed using the one-way ANOVA test at a significance level of 5%.

**Group K15+PTN:** the glide path was created with a #15 K-type hand file up to the WL, and instrumentation was performed with instruments X1 (17/.04) and X2 (25/.06) of the ProTaper Next system;

**Group PG+PTN:** the glide path was created with the ProGlider instrument up to the WL, and instrumentation was performed with instruments X1 (17/.04) and X2 (25/.06) of the ProTaper Next system;

**Group K15+PTG:** the glide path was created with a #15 K-type hand file up to the WL, and instrumentation was performed with instruments S1 (18/.02), S2 (20/.04), F1 (20/.07) and F2 (25/.08) of the ProTaper Gold system; and

**Group PG+PTG:** the glide path was created with the ProGlider instrument up to the WL, and instrumentation was performed with instruments S1 (18/.02), S2 (20/.04), F1 (20/.07) and F2 (25/.08) of the ProTaper Gold system.

All of the materials used in the study were purchased at a dental materials store in the city of São Paulo, SP, Brazil. Before the chemical-surgical preparation, the tooth roots were covered with condensation silicone (Zetaplus; Zhermack, Badia Polesine, Italy) to ensure that the irrigating solution would be maintained inside the canal, and not leak through the apical foramen. The speed and torque settings employed were those recommended by the manufacturer for each system, and were pre-set in the X Smart Plus endodontic motor (Dentsply-Mailefer), as follows: ProGlider, 200 rpm and 2 Ncm; ProTaper Next, 300 rpm and 2 Ncm for all instruments; ProTaper Gold, 250 rpm and 3 Ncm for instruments S1 and SX, 250 rpm and 1 Ncm for instrument S2, 250 rpm and 1.5 Ncm for instrument F1 and, lastly, 250 rpm and 2 Ncm for instrument F2.

During the shaping procedure, all the instruments were applied using 3 gentle in-and-out motions. The canals were irrigated at each instrument change with 5 mL of 2.5% sodium hypochlorite using a disposable syringe and a 27-gauge Endo-Eze irrigator tip needle (Ultradent, South Jordan, USA), placed 1 mm short of the WL. After irrigation, a #10 K-type manual file was introduced up to the apical foramen to maintain foraminal patency. A final rinse with 5 mL of 17% EDTA was followed by a 5-mL rinse with distilled water. In the groups where PTN instruments were used, irrigation was supplemented with 2.5% sodium hypochlorite in order to maintain an irrigant volume equivalent to that used in the groups where PTG instruments were used.

All the instruments were used on a single specimen and then discarded. All of the experimental procedures were performed by a single endodontics specialist (B.N.V.).

### Micro-CT scanning and image analysis

The teeth were submitted to a micro-CT scan (SkyScan 1176; Bruker, Kontich, Belgium) before and after chemical-surgical preparation, using the settings of 90 kV, 278 mA, 360° rotation, and a 0.5° rotation step to produce a voxel size of 17.42 mm. After acquisition of 2D images, NRecon v. 1.6.10.4 software (Bruker) was used to reconstruct the cross-sections, using 800 to 900 sections per specimen.

The pre- and post-operative images were reconstructed, and then Data Viewer v. 1.5.1 software (Bruker) was used to register the image sets and align the two reconstructions geometrically. CTAn v. 1.14.4 software (Bruker) was used to calculate the quantitative parameters and build the models. CTVol v. 2.2.1.0 software (Bruker) was used to visualize and produce the 3D images.

The volume of interest in each specimen was considered as that measured from the furcation region up to the apex of the mesial roots of the mandibular molars. Image binarization was performed to segment the endodontic space and root dentin. Canals were analyzed for volumetric variation, untouched surface, and canal transportation. The volume increase percentage (%D) was determined using the following formula:

%Δ=([A−B]/B)×100 , where *%Δ* is the percentage of volumetric variation, A is the volume observed after instrumentation, and B is the volume observed before instrumentation.

The untouched surface variable was calculated by subtracting the number of voxels removed after instrumentation from the total number of surface voxels before instrumentation, using the following formula:

% US = US X 100/ IS  , where *%US* is the untouched surface percentage, US is the untouched surface, and IS is the initial surface.

Canal transportation was assessed using the following formula:

$D^{2}={\left(x_{1}-x_{2}\right)}^{2}+{\left(y_{1}-y_{2}\right)}^{2}+{\left(z_{1}-z_{2}\right)}^{2}$ , where x_1_, y_1_ and z_1_ are the coordinates of the center of gravity observed in each cut before instrumentation, and x_2_, y_2_ and z_2_ are the coordinates of the center of gravity observed in each cut after instrumentation.

The canal transportation data were transferred to Graph Maker online software (Plotly, Montreal Island, Canada), and 3D graphs illustrating root canal transportation were created by drawing a line connecting the centers of gravity of the several root sections of each specimen. Next, the pre- and postoperative lines thus obtained for each canal were superimposed to evidence the level of canal transportation.

### Statistical analysis

Two-way analysis of variance (ANOVA), complemented by Tukey’s test for multiple comparisons, was used to determine the differences between the study groups with respect to the canal volume, untouched surface percentage, and canal transportation variables. GraphPad Prism 7 software (GraphPad Software, Boston, USA) was used in the analyses. The level of significance was set at p < 0.05.

## RESULTS

**Figure 1:**
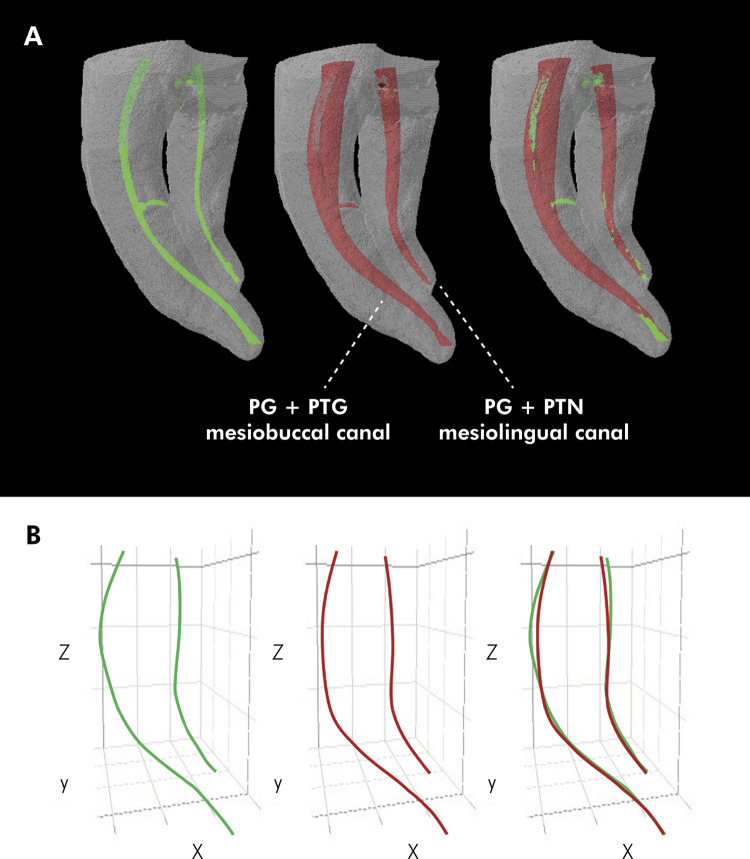
A: Images representative of the three-dimensional models constructed from the preoperative (green) and postoperative (red) micro-CT images, and superimposition of one onto the other, for groups PG+PTG (mesiobuccal canal) and PG+PTN (mesiolingual canal), showing the behavior of the initial volume, final volume, and volumetric variation variables. B: Lines representing the root canal trajectory constructed by connecting the x, y, and z coordinates of the centers of gravity of the root sections analyzed before and after instrumentation, showing the behavior of the canal transportation variable. Group PG+PTG: glide path creation performed with the ProGlider instrument, and instrumentation, with the ProTaper Gold system. Group PG+PTN: glide path creation performed with the ProGlider instrument, and instrumentation, with the ProTaper Next system.

**Figure 2:**
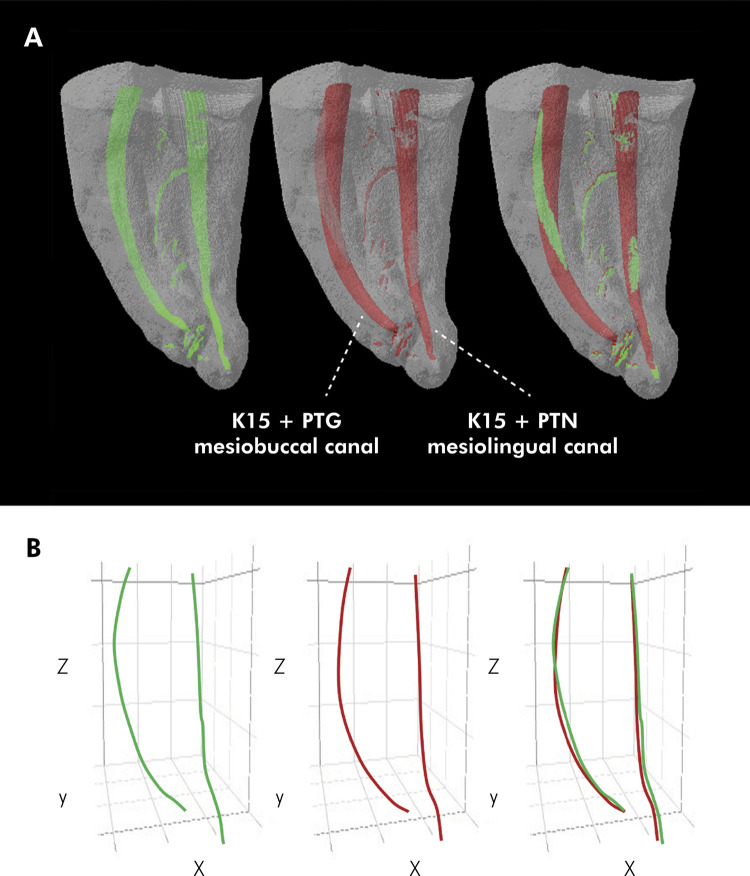
A: Images representative of the three-dimensional models constructed from the preoperative (green) and postoperative (red) micro-CT images, and superimposition of one onto the other, for groups K15+PTG (mesiobuccal canal) and K15+PTN (mesiolingual canal), showing the behavior of the initial volume, final volume, and volumetric variation variables. B: Lines representing the root canal trajectory constructed by connecting the x, y, and z coordinates of the centers of gravity of the root sections analyzed before and after instrumentation, showing the behavior of the canal transportation variable. Group K15+PTG: glide path creation performed with a #15 K-type hand file, and instrumentation, with the ProTaper Gold system. Group K15+PTN: glide path creation performed with a #15 K-type hand file, and instrumentation, with the ProTaper Next system.

Figures 1 and 2 show representative images of the three-dimensional models constructed from the pre- and postoperative micro-CT scans for the study groups, showing the behavior of the initial volume and final volume variables, as well as the superimposition of one onto the other. They also show lines representing the root canal trajectories constructed by connecting the x, y, and z coordinates of the centers of gravity of the analyzed sections before and after instrumentation, as well as the superimposition of one onto the other to evidence the behavior of the canal transportation variable.

**Figure 3:**
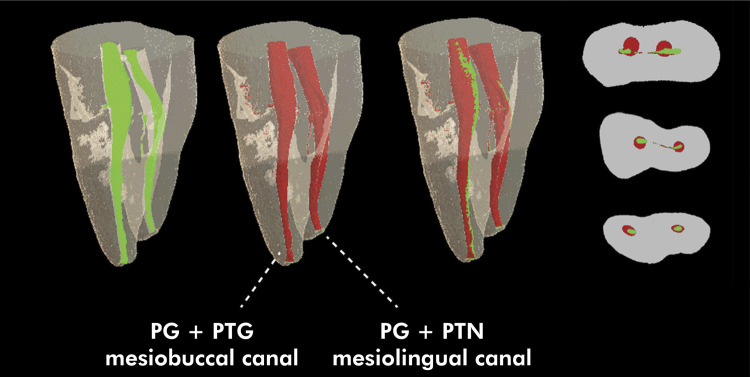
Images representative of the three-dimensional models constructed from the preoperative (green) and postoperative (red) micro-CT images, and superimposition of one onto the other, as well as 2D sections representative of the cervical, middle, and apical thirds of the root canal, for groups PG+PTG (mesiobuccal canal) and PG+PTN (mesiolingual canal), showing the behavior of the untouched walls variable.

**Figure 4:**
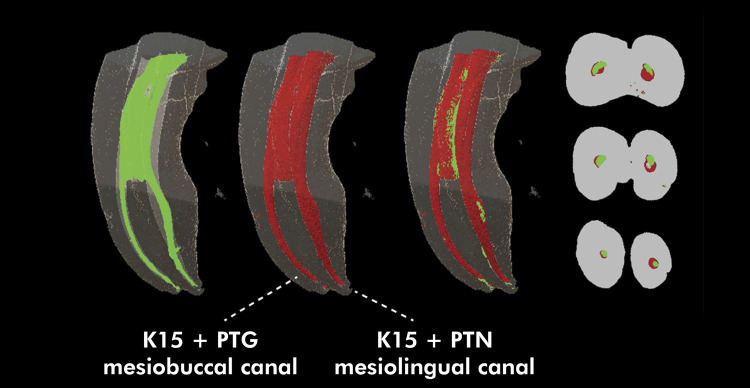
Images representative of the three-dimensional models constructed from the preoperative (green) and postoperative (red) micro-CT images, and superimposition of one onto the other, as well as 2D sections representative of the cervical, middle, and apical thirds of the root canal, for groups K15+PTG (mesiobuccal canal) and K15+PTN (mesiolingual canal), showing the behavior of the untouched walls variable.

Figures 3 and 4 show representative images of the three-dimensional models constructed from the pre- and postoperative micro-CT scans for the study groups, as well as the superimposition of one onto the other, and representative images of the 2D sections from the cervical, middle and apical root thirds, evidencing the behavior of the untouched walls variable.

Tables 1, 2, and 3 show the data regarding root canal volume increase (volumetric variation), rate of untouched walls, and root canal transportation, respectively, observed after using the glide path instruments and the instrumentation systems tested. Table 4 shows data regarding the rate of untouched walls after using the glide path instruments tested, irrespective of the instrumentation system. There was no significant difference among groups with respect to initial volume, final volume, volumetric variation (Table 1) or root canal transportation (Table 3), either for the cervical, middle or apical root thirds, or for the entire root canal (p > 0.05).

**Table 1:** Mean and standard deviation values of the initial and final root canal volumes (mm3), and volumetric variation (mm3 and %), observed after use of the instrumentation systems and glide path creation instruments.

Region	ProTaper next		ProTaper gold	

	File # 15	ProGlider	File # 15	ProGlider
Initial canal volume (mm^3^)				
Total	1.586 ± 0.819	2.056 ± 0.607	1.446 ± 0.358	1.769 ± 0.745
Cervical third	0.909 ± 0.447	1.172 ± 0.282	0.776 ± 0.253	0.864 ± 0.237
Middle third	0.508 ± 0.366	0.673 ± 0.269	0.455 ± 0.118	0.611 ± 0.308
Apical third	0.168 ± 0.073	0.217 ± 0.090	0.217 ± 0.103	0.293 ± 0.233
Final canal volume (mm^3^)				
Total	2.600 ± 1.003	3.280 ± 0.969	3.395 ± 1.740	2.937 ± 1.092
Cervical third	1.532 ± 0.511	1.882 ± 0.525	1.845 ± 0.927	1.529 ± 0.438
Middle third	0.800 ± 0.413	1.020 ± 0.324	1.110 ± 0.609	0.984 ± 0.383
Apical third	0.270 ± 0.148	0.388 ± 0.174	0.452 ± 0.253	0.460 ± 0.239
Canal volume increase (mm^3^)				
Total	1.015 ± 0.728	1.223 ± 0.648	1.949 ± 1.628	1.168 ± 0.513
Cervical third	0.632 ± 0.400	0.710 ± 0.394	1.069 ± 0.889	0.665 ± 0.277
Middle third	0.291 ± 0.256	0.347 ± 0.176	0.655 ± 0.579	0.373 ± 0.159
Apical third	0.102 ± 0.113	0.171 ± 0.127	0.235 ± 0.192	0.217 ± 0.213
Canal volume increase (%)				
Total	87.688 ± 75.827	62.154 ± 37.115	142.183 ± 109.049	71.949 ± 33.569
Cervical third	98.334 ± 86.455	62.196 ± 37.588	163.331 ± 150.707	78.668 ± 32.798
Middle third	89.554 ± 92.210	58.787 ± 44.704	152.058 ± 124.470	72.588 ± 38.908
Apical third	80.652 ± 59.908	92.866 ± 75.922	96.774 ± 68.719	80.969 ± 54.202

**Table 2:** Mean and standard deviation values of the untouched canal wall percentages observed after use of the instrumentation systems and glide path creation instruments.

Untouched canal walls (%)	ProTaper next		ProTaper gold	

	File # 15	ProGlider	File # 15	ProGlider
Total	37.428 ± 13.844	28.702 ± 14.705	30.572 ± 10.801	29.560 ± 12.758
Cervical third	38.284 ± 20.112	30.867 ± 11.218	30.654 ± 11.732	32.435 ± 10.544
Middle third	29.859 ± 21.645	27.034 ± 17.719	26.990 ± 14.903	27.230 ± 12.369
Apical third	36.559 ± 14.680 ^A^	20.630 ± 17.486 ^B^	29.730 ± 14.749 ^A^	25.940 ± 16.206 ^B^

Different superscript letters in the same row indicate a statistically significant difference between groups (p < 0.05).

**Table 3:** Mean, standard deviation, and minimum and maximum values of the root canal transportation (mm) observed after use of the instrumentation systems and glide path creation instruments.

Canal transportation (mm)	ProTaper next		ProTaper gold	

	File # 15	ProGlider	File # 15	ProGlider
Total	0.078 ± 0.053	0.068 ± 0.028	0.080 ± 0.038	0.077 ± 0.055
	0.020 – 0.163	0.036 – 0.139	0.026 – 0.149	0.044 – 0.243
Cervical third	0.125 ± 0.107	0.084 ± 0.046	0.109 ± 0.067	0.108 ± 0.112
	0.015 – 0.328	0.043 – 0.179	0.023 – 0.239	0.038 – 0.456
Middle third	0.064 ± 0.046	0.067 ± 0.031	0.079 ± 0.049	0.063 ± 0.033
	0.022 – 0.175	0.025 – 0.148	0.020 – 0.183	0.021 – 0.124
Apical third	0.045 ± 0.024	0.053 ± 0.028	0.053 ± 0.022	0.059 ± 0.037
	0.011 – 0.080	0.014 – 0.093	0.029 – 0.0871	0.006 – 0.151

**Table 4:** Mean and standard deviation values of the untouched canal wall percentages observed after use of the glide path creation instruments, irrespective of the instrumentation system used.

Untouched canal walls (%)	K-type file # 15	ProGlider
Total	33.999 ± 12.637	29.130 ± 13.470
Cervical third	34.469 ± 16.567	31.651 ± 10.676
Middle third	28.424 ± 18.232	27.132 ± 14.944
Apical third	33.144 ± 14.807 ^A^	23.285 ± 16.709 ^B^

Different superscript letters in the same row indicate a statistically significant difference between groups (p < 0.05).

When only the instrument used to create the glide path is taken into account, the percentage of untouched walls in the apical third was significantly higher in the groups where a #15 K-type hand file was used than in those where the ProGlider instrument was used (p < 0.05, Tables 2 and 4). In the other regions, there was no significant difference between these instruments with respect to this variable.

## Discussion

The null hypothesis of the present study was partially rejected, in that the instrument chosen to create the glide path had a direct influence on the observed rates of untouched walls in the apical third of mesial canals of severely curved mandibular molars, irrespective of the instrumentation system used.

Curved canals pose a greater challenge to root canal preparation, since certain areas of these root canals are eventually left untouched by the instruments.^28^ Selection of the teeth included in the present study focused on standardizing specimens for root curvature. Accordingly, mandibular molars were chosen, because their mesial roots often present curvatures of about 60%.^29^ Because the literature on the behavior of the variables studied herein regarding severely curved specimens is scarce, teeth with curvatures ranging from 40° to 68° (average of 54°) were selected. Another relevant aspect associated with severe curvatures is the greater susceptibility to fracture of rotary NiTi instruments during preparation of root canals with this feature.^30,31^ In the present study, five teeth were promptly replaced due to fractured rotary instruments, namely two ProGlider instruments, two ProTaper Next X2 instruments, and one ProTaper Gold S2 instrument. Severe curvature of the root canals may have contributed to the fractures observed in this study, particularly of the two ProTaper Next X2 instruments and the ProTaper Gold S2 file. Previous studies^22,23,30,31^have demonstrated that larger caliber instruments are more susceptible to fatigue fracture. Therefore, there is an association of two risk factors, severe curvatures and instrument size. Another aspect to be considered is the alloy used in the manufacturing of the instruments. In ProTaper Next instruments, manufactured with M-Wire alloy, the austenite phase of the NiTi alloy predominates, leading to low instrument flexibility. In contrast, failure of the two ProGlider instruments may have been associated with locking of the instrument in the more apical portions of the root canal, thus causing torsional fracture.

Use of the progressively tapered (2%–8%) ProGlider instrument provides a canal pre-enlargement wider than that provided by a #15 K-type file. In the present study, this effect seemed to have favored the sequential use of the subsequent rotary instruments, resulting in an improved apical third preparation. According to Lopes et al.,^32^ greater enlargement of the canal during glide path creation with a mechanized instrument leads to less friction of subsequent instruments against the entire extension of the root canal walls during the shaping stage.

Root canal transportation is a relatively common procedural error observed during instrumentation^33^, and can lead to inadequate disinfection, overfilling, and underfilling of the root canal.^34^ This variable can be assessed by measuring the displacement of the surgical canal’s center of gravity in relation to the anatomical canal’s center of gravity, in absolute values. Current concepts of root canal shaping posit that glide path creation with mechanized instruments can minimize the occurrence of procedural errors during root canal treatment,^35^ and that manual steel instruments can effect comparatively more significant deviations of the root canal.^19^ According to Alves et al.,^36^ mechanized glide path creation helps preserve the original anatomy of the root canal, provides comparatively more centered preparations, and is associated with lower levels of transportation in curved canals.^37^

Several studies have used micro-CT to assess root canal transportation;^18-20^ however, the analyses they performed involved only a limited number of root canal sections. In contrast, the methodology of the present study used a three-dimensional analysis that made it possible to measure deviation of the center of gravity along the entire canal, in all the cross-sections provided by the micro-CT scan. This data was used to build 3D models that allowed visualizing canal configuration before and after preparation. Gagliardi et al.^4^ also assessed the ProTaper Gold and ProTaper Next systems, but they used less severely curved canals, and did not evaluate the influence of the glide path creation procedure. In their study, the authors observed that the instruments made of heat-treated NiTi alloys did not cause canal transportation, as opposed to the instrument made of a conventional NiTi alloy, which did, thus corroborating the results of the present study.

In the present study, no significant differences were observed among the study groups regarding canal transportation, either in the assessment of the entire canal or in the assessment of its thirds, irrespective of the instrument used for glide path creation. This result can be attributed to the rotary NiTi instruments’ being capable of preparing the root canal while maintaining its original curvature, even in extremely curved canals.^24,38,39^ This concept is even more pertinent when preparation is conducted with instruments submitted to specific heat treatments that impart greater strength and flexibility, such as those of the ProTaper Gold and ProTaper Next systems.^2^ It can be assumed that use of a mechanized system for glide path creation allows subsequent shaping instruments to be effective, even when a smaller number of “pecking” motions are performed to reach the WL. Hence, it can also be assumed that the risk of an ill-directed instrumentation or of deviations occurring when such a system is used is lower, since unnecessary dentin wear is avoided.^34,40^

The findings of the present study suggest that both instrumentation systems provide adequate shaping of the root canal; however, their extrapolation to clinical practice should be done with caution, considering the anatomical variability among patients, and the fact that the micro-CT assessment method cannot be applied in vivo. Furthermore, considering that the use of root canal shaping instruments could correct occasional glide path imperfections, future studies performing both assessments— immediately after glide path creation, and then again after root canal shaping—are warranted to determine the individual contributions of each stage of the process.

## Conclusions

It was concluded that use of the ProGlider mechanized instrument for glide path creation provided an endodontic preparation with a smaller rate of untouched walls in the apical region than use of a #15 K-type manual file for this purpose. There was no significant difference between the ProTaper Gold and ProTaper Next instrumentation systems regarding volumetric variation, untouched walls or root canal transportation.
